# Incidence Rates for Tuberculosis Among HIV Infected Patients in Northern Tanzania

**DOI:** 10.3389/fpubh.2019.00306

**Published:** 2019-10-24

**Authors:** Edson W. Mollel, Werner Maokola, Jim Todd, Sia E. Msuya, Michael J. Mahande

**Affiliations:** ^1^Department of Epidemiology and Biostatistics, Institute of Public Health, Kilimanjaro Christian Medical University College, Moshi, Tanzania; ^2^Northern Zone Blood Transfusion Center, Moshi, Tanzania; ^3^National AIDS Control Program, Dar es Salaam, Tanzania; ^4^Department of Population Health, London School of Hygiene and Tropical Medicine, London, United Kingdom; ^5^Department of Community Health, Institute of Public Health, Kilimanjaro Christian Medical University College, Moshi, Tanzania; ^6^Department of Community Medicine, KCMC Hospital, Moshi, Tanzania

**Keywords:** tuberculosis, HIV, Tanzania, incidence rates, sub-Saharan Africa

## Abstract

**Background:** HIV and tuberculosis (TB) are leading infectious diseases, with a high risk of co-infection. The risk of TB in people living with HIV (PLHIV) is high soon after sero-conversion and increases as the CD4 counts are depleted.

**Methodology:** We used routinely collected data from Care and Treatment Clinics (CTCs) in three regions in northern Tanzania. All PLHIV attending CTCs between January 2012 to December 2017 were included in the analysis. TB incidence was defined as cases started on anti-TB medications divided by the person-years of follow-up. Poisson regression with frailty models were used to determine incidence rate ratios (IRR) and 95% confidence intervals (95% CI) for predictors of TB incidences among HIV positive patients.

**Results:** Among 78,748 PLHIV, 405 patients developed TB over 195,296 person-years of follow-up, giving an overall TB incidence rate of 2.08 per 1,000 person-years. There was an increased risk of TB incidence, 3.35 per 1,000 person-years, in hospitals compared to lower level health facilities. Compared to CD4 counts of <350 cells/μl, a high CD4 count was associated with lower TB incidence, 81% lower for a CD4 count of 350–500 cells/μl (IRR 0.19, 95% CI 0.04–0.08) and 85% lower for those with a CD4 count above 500 cells/μl (IRR 0.15, 95% CI 0.04–0.64). Independently, those taking ART had 66% lower TB incidences (IRR 0.34, 95% CI 0.15–0.79) compared to those not taking ART. Poor nutritional status and CTC enrollment between 2008 and 2012 were associated with higher TB incidences IRR 9.27 (95% CI 2.15–39.95) and IRR 2.97 (95% CI 1.05–8.43), respectively.

**Discussion:** There has been a decline in TB incidence since 2012, with exception of the year 2017 whereby there was higher TB incidence probably due to better diagnosis of TB following a national initiative. Among HIV positive patients attending CTCs, poor nutritional status, low CD4 counts and not taking ART treatment were associated with higher TB incidence, highlighting the need to get PLHIV on treatment early, and the need for close monitoring of CD4 counts. Data from routinely collected and available health services can be used to provide evidence of the epidemiological risk of TB.

## Introduction

Tuberculosis (TB) is a disease caused by *Mycobacteria tuberculosis*, which can be latent in humans for a long time without clinical symptoms. Active TB can present as Pulmonary Tuberculosis (PTB) or Extra-Pulmonary Tuberculosis (EPTB), with cardinal features of fever, productive cough, hemoptysis, and weight loss, though the presentation among HIV infected individuals is often atypical. Several factors have been associated with an increased risk of TB incidence, such as poverty, malnutrition, and overcrowding, but the risk of active TB is 16–27 times higher in people living with HIV (PLHIV) compared to those who are HIV negative (1). This is due to the impaired and lowered innate and passive immunity against TB among PLHIV (2), increasing the risk of getting a new TB infection (3) and of progression from latent TB to active TB (3). New TB infections, rather than reactivation, account for 88% of new TB cases among PLHIV (4).

The risk of TB for PLHIV is high soon after sero-conversion (5), and continues to increase with depletion of CD4 count (6). The CD4 count is a diagnostic and/or prognostic marker that normally measures the number of CD4 expressing T-cells (also known as T helper cells). But the risk of TB among PLHIV decreases after starting anti-retroviral therapy (ART) (53).

TB incidence has been falling since 2013, by 2% globally and by 4% in Africa. However, in 2017, 10 million (range 9–11.1 million) new cases of TB were reported worldwide, of which 25% occurred in Africa and 87% in 30 high TB burden countries (Tanzania included) (7). Of the cases, 90% were >15 years of age, 64% were males and 9% were HIV positive (7). The reported cases include only 51% of the estimated 920,000 new TB cases among PLHIV. Of the 1.5 million people enrolled at Care and Treatment Clinics (CTCs) in 2017, 8% were diagnosed with TB in the same year. Africa accounted for 72% of all HIV associated TB cases in 2017 (7). The End TB Strategy has set a reduction target of 80% in TB incidence (new cases per 100,000 population per year), compared to the level in 2015 (7). Tanzania is one of the High TB Burden Countries, and one of the High TB/HIV Burden Countries. In Tanzania, it is estimated that of 154,000 (range 73,000–266,000) new cases of TB in 2017, 31% (48,000 [31,000–69,000]) were also HIV positive (7). But with only 93% of TB patients in Tanzania having test results for HIV, of which 36% were co-infected with HIV, the true burden of TB among HIV positive people could be underestimated. TB has been the leading cause of death among HIV positive individuals (7), so a close monitoring of its occurrence in this subgroup of people is extremely important.

Several factors have been associated with TB incidence among HIV positive individuals including limited functional status, very low CD4 count (<50 cells/μl) (53), anemia, inappropriate vaccinations, cigarette smoking, households with a family size of 3 to 4 people, a lower social class, non-adherence to drugs and severe immunosuppression (8).

Several interventions have been implemented to try to reduce the incidence of TB in Tanzania's general population and among HIV positive individuals. As some patients may present with subclinical TB (9), WHO recommended active TB screening (intensified case finding) for all PLHIV, and infection control (10). In 2010, a gradual implementation of Genexpert MTB/RIF for the early diagnosis of TB among all TB suspects started, and was scaled up in 2013 (11). This test was initially only for HIV positive patients or for those with recurring TB. In 2011, the country introduced Isoniaziad Preventive Therapy (IPT) among PLHIV (12), which appears to be effective at reducing TB incidence (13). Recently, WHO is recommending a “test and treat” policy which requires all individuals being diagnosed as HIV positive to be put on ARVs immediately (14). Data from CTCs in Northern Tanzania provides an opportunity to track the incidence of TB among HIV patients through this spectrum of different intervention programs.

It is important to study TB incidence rates among PLHIV in Tanzania and compare it with the estimated global TB incidence rate that has been calculated by WHO (7). This can guide clinicians and policy makers on interventions and practices to improve health outcomes, and help to develop preventive measures to reduce the magnitude of the problem. This study determined the predictors and TB incidences among HIV positive patients since enrolment at Care and Treatment Centers (CTCs) after a follow up period of 6 years (January 2012 to December 2017), in the Northern part of Tanzania. Hence providing a big picture of the effects of several interventions that have been implemented over the years.

## Methodology

### Study Design and Settings

This was a retrospective cohort study which included data which have been routinely collected from patients attending CTCs from 1st January 2012 to 31st December 2017 in the Arusha, Kilimanjaro and Tanga regions. Both public and private CTCs were included, categorized as hospitals (at district level and above), health centers and dispensaries. At every visit, all HIV patients attending a CTC have a regular check-up, and a screening for opportunistic infections. The screening for TB follows WHO recommendations through the assessment of symptoms and signs. All these positive on the screening symptoms (either showing a productive cough, persistent low grade fever, night sweats, or weight loss) have to undergo further testing. This can be done using Genexpert MTB/RIF, or sputum microscopy at centers that have no Genexpert MTB/RIF. Genexpert MTB/RIF is a molecular diagnostic tool used for diagnosis of *M. tuberculosis* (MTB) and Resistance of these strains to Rifampicin (RIF). Sputum from the patient is mixed with Genexpert MTB/RIF buffer solution and is shaken and incubated for 5–10 min, before being pipetted into cartridge of Genexpert MTB/RIF for computer assisted diagnosis. Those diagnosed with TB are given anti-TB medication.

### Study Population and Data Definitions

All patients (above 15 years of age) who were HIV positive and attended one of 489 CTCs in the regions of Arusha, Kilimanjaro, and Tanga during the period of 1st January 2012 to 31st December 2017 were included in this study. Those who already had a TB diagnosis and/or were on TB treatment at the time of their first visit to the CTC were excluded, whilst those treated for TB before the start of the study duration were not excluded. The start time was taken to be 1st January 2012, or the date of first enrollment at the CTC if enrollment was after 1st January 2012. End time was defined as whichever came first among the following; the date there were last seen at a CTC, the date of death, the date of the first TB incidence, or 31st December 2017. A TB diagnosis was defined as being started on anti-TB medications after being screened for TB during a visit to a CTC, regardless of the method used to confirm a TB diagnosis. The following predictor variables were collected; age, sex, marital status, geographical location, baseline weight, baseline HIV WHO clinical stage, use of ARV, use of IPT, functional status, ARV adherence status, CTC enrollment year, type of ARV regimen, and baseline CD4 counts.

### Data Analysis

Data were de-identified and analyzed using a statistical software package, STATA 15. After data cleaning, categorical data were summarized as frequencies and percentages. Continuous variables were summarized using their median and interquartile range (IQR) or by using their mean and standard deviation.

Incidence rates, and 95% confidence intervals (95%CI), for each level of independent variable were determined, as the number of newly diagnosed TB cases over the person-years at risk. Health facilities were used as a cluster variable, and Analysis of Variance (ANOVA) was used to compare aggregate rates of TB incidences by: health facility levels, health facility types (dispensaries, health centers, and hospitals), health facility ownership (private and public ownership) and regions (Arusha, Kilimanjaro, and Tanga). A Poisson regression model with frailty to adjust for the clustering at health facilities was used to obtain incidence rate ratios (IRR) for TB, and 95% CI for socio-demographic and clinical characteristics of the patients. Crude incidence rate ratios were then adjusted for other independent factors for TB.

### Ethical Clearance

Ethical clearance was obtained from Kilimanjaro Christian Medical University College Research and Ethical Committee (Ethical clearance certificate number 2286). Permission from the Ministry of Health—Tanzania and NACP (National AIDS Control Program) authority to conduct the study was obtained. All patients' privacy and confidentiality were strictly observed throughout the study.

## Results

The study included 78,748 HIV-positive patients who were followed up for 195,296 person-years, with 405 patients recorded as having had a new episode of TB during the follow up, giving an incidence of TB of 2.08 (95% CI 1.88–2.29) per 1,000 person-years (Table 1). Looking at ages, the highest incidence rates for TB, 2.45 per 1,000 person-years (95% CI 2.01–2.99), were in patients aged 35–44 years of age. The TB incidence in males (Incidence = 3.70 per 1,000 person-years, 95% CI 3.21–4.27) was higher than in females (Incidence = 1.50 per 1,000 person-years, 95% CI 1.31–1.72). Those who were divorced and those from Arusha region had higher TB incidences with incidence of 2.58 (95% CI 1.95–3.41) and 2.30 (95% CI of 1.74–3.12) per 1,000 person-years, respectively, than others (Table 1).

**Table 1:** Incidence rates for TB by socio-demographic characteristics at enrolment into HIV services in 78,748 patients in three regions of Tanzania.

**Characteristics**	**Number of PLHIV**	**Number of TB cases**	**Person-years (in 1,000)**	**Incidence rate** **per 1,000** **(95% CI)**
**Overall**	78,748	405	195.1	2.08 (1.88–2.29)
**Age**				
15–24	8,453	28	13	2.15 (1.48–3.11)
25–34	21,150	98	40	2.45 (2.01–2.99)
35–44	26,281	143	68.8	2.08 (1.77–2.45)
45–55	15,343	92	47.4	1.94 (1.58–2.38)
Above 55	7,521	44	26	1.70 (1.26–2.28)
**Sex**				
Male	21,983	188	50.7	3.70 (3.21–4.27)
Female	56,764	217	144.4	1.50 (1.31–1.72)
**Marital status**				
Cohabiting	1,013	4	2.6	1.51 (0.57–4.03)
Divorced	7,335	49	19	2.58 (1.95–3.41)
Married	39,151	191	100.5	1.90 (1.65–2.19)
Single	19,969	111	48.4	2.29 (1.90–2.76)
Widow/widower	5,670	26	15.2	1.71 (1.17–2.51)
**Region**				
Arusha	12,348	45	19.3	2.30 (1.74–3.12)
Kilimanjaro	26,133	134	66	2.03 (1.71–2.41)
Tanga	40,267	226	109.9	2.06 (1.81–2.34)
**Year of follow up**				
2012	44,673	65	30.3	2.15 (1.68–2.74)
2013	12,288	81	30.8	2.63 (2.12–3.27)
2014	12,117	68	34	2.00 (1.58–2.54)
2015	10,563	46	36.9	1.25 (0.93–1.67)
2016	8,728	56	38.2	1.47 (1.13–1.91)
2017	6,208	89	25.1	3.55 (2.88–4.37)

HIV patients with markers of lower immunity or advanced disease (HIV stage 3&4, CD4 < 350 cells/μl, lower weight and poorer nutritional status) had higher TB incidence than others (Table 2). Patients with a severely poor nutritional status had a TB incidence rate of 47.74 per 1,000 person-years (95% CI 26.44–86.21), while those with a moderately poor nutritional status had a TB incidence rate of 9.53 per 1,000 person-years (95% CI 7.12–12.77), and those with an adequate nutritional status had a TB incidence rate of 1.73 per 1,000 person-years (95% CI 1.54–1.95) (Table 2). Higher TB incidence rates was found in those who were bedridden (Incidence = 32.20 per 1,000 person-years, 95% CI 24.89–41.65) and those who were ambulatory (Incidence = 31.06 per 1,000 person-years, 95% CI 18.99–54.14), compared to those who were working (Incidence = 1.73 per 1,000 person-years, 95% CI 1.55–1.93) (Table 1).

**Table 2:** Incidence rates for TB by clinical characteristics at enrolment into HIV services in 78,748 patients in three regions of Tanzania.

**Characteristics**	**Number of PLHIV**	**Number of TB cases**	**Person-years per 1,000**	**Incidence rate** **per 1,000** **(95% CI)**
**Body weight**				
Below 40 kg	5,276	68	7.7	8.88 (7.00–11.27)
40–60 kg	44,135	263	100.9	2.61 (2.31–2.94)
Above 60 kg	23,680	49	67.2	0.73 (0.55–0.96)
**HIV WHO stage**				
Stage 1	17,347	18	28.4	0.63 (0.40–1.01)
Stage 2	17,390	28	37.2	0.75 (0.52–1.09)
Stage 3	30,505	223	91.5	2.44 (2.14–2.78)
Stage 4	11,165	127	31.9	3.98 (3.34–4.73)
**CD4 categories**				
Below 350	7,808	42	12.6	3.33 (2.46–4.51)
350–500	2,561	6	6.5	0.93 (0.42–2.06)
Above 500	3,098	4	9	0.44 (0.17–1.19)
**Use of IPT**				
No	78,504	405	193.7	2.09 (1.90–2.31)
Yes	244	0	1.5	0
**Ever use ART**				
No	5,428	72	17.9	4.03 (3.20–5.08)
Yes	73,320	333	177.3	1.88 (1.69–2.09)
**Functional status**				
Bedridden	2,647	58	1.8	32.20 (24.89–41.65)
Ambulatory	340	14	0.4	31.06 (18.99–54.14)
Working	74,921	327	189	1.73 (1.55–1.93)
**CTC enroll year**				
2003–2007	12,897	69	59.7	1.16 (0.91–1.46)
2008–2012	32,057	131	83.5	1.57 (1.32–1.86)
2013–2017	33,794	205	51.9	3.95 (3.44–4.53)
**Nutritional status**				
Ok	50,727	278	160.5	1.73 (1.54–1.95)
Moderate	3,603	45	4.7	9.53 (7.12–12.77)
Severe	218	11	0.2	47.74 (26.44–86.21)
**ARV regimen**				
First line	57,802	293	173.5	1.69 (1.51–1.89)
Second line	745	15	5.1	2.95 (1.78–4.89)
Others	512	0	0.4	–

Analysis of Variance (ANOVA) was used to compare rates of TB incidences across the following cluster variables: facility types (dispensaries, health centers and hospitals), facility ownership (private and public ownership) and regions (Arusha, Kilimanjaro, and Tanga). There was a significant increased risk of TB incidence in hospitals (3.35 per 1,000 person-years) compared to TB incidences in the health centers (1.28 per 1,000 person-years) and dispensaries (1.36 per 1,000 person-years), *p*-value 0.0306. There were no statistically significant differences in TB incidences for the cluster variables of facility ownership and region (Table 3).

**Table 3:** Comparison of cluster level's TB rates per 1,000 person-years.

**Cluster variables**	**Number of TB cases**	**Number of cluster variables with at least 1 TB case**	**Mean/average TB cases per cluster variable**	**Average TB rates per 1,000 person-years per cluster variable**	***p*-value (ANOVA) comparing TB rates for cluster variables**
**Facility types**					
Dispensary	12	38	0.32	1.36	0.0306
Health centers	131	70	1.87	1.28	
Hospitals	262	32	8.19	3.35	
Overall	405	140	2.89	1.77	
**Facility ownership**					
Private	50	34	1.47	2.03	0.6632
Public	355	106	3.35	1.69	
Overall	405	140	2.89	1.77	
**Region**					
Arusha	45	39	1.15	1.86	0.6754
Kilimanjaro	134	43	3.12	1.35	
Tanga	226	58	3.9	2.03	
Overall	405	140	2.89	1.77	

After performing a multilevel analysis and controlling for health facilities as clusters, several factors were significantly related to an increased TB incidence among HIV positive patients such as the year of enrollment at a CTC, those enrolled between 2008 and 2012 had an IRR of 1.51 (95% CI 1.12–2.03) while those enrolled between 2013 and 2017 had an IRR of 4.05 (95% CI 3.04–5.39). Moderate and severe nutritional status were also significantly associated with TB incidence among HIV patients, with an IRR of 6.94 (95% CI 4.92–9.80) and 28.05 (95% CI 15.09–52.16), respectively. The use of second line ARVs had an IRR of 1.75 (95% CI 1.04–2.97). The study found that several factors were protective against developing new TB among HIV patients, including being female and having CD4 count between 350 cells/μl to 500 cells/μl both of which were protective by 58%, with IRRs of 0.42 (95% CI 0.34–0.50) and 0.42 (95% CI 0.21–0.87), respectively. Using ARVs was protective by 57%, IRR 0.43 (95% CI 0.33–0.55). CD4 counts above 500 cells/μl was protective by 84%, IRR 0.16 (95% CI 0.07–0.42) and having a working functional status, were even more protective, by 95%, IRR 0.05 (95% CI 0.04–0.07) (Table 4).

**Table 4:** Poisson regression with multilevel analysis of the determinants of TB incidence in Northern Tanzania.

**Characteristics**	**Survival status**		**Crude hazard ratio (95%C)**	**Adjusted hazard ratio (95%CI) adjusting only for clusters**	**AHR (95% CI) adjusting for other factors and for clusters**
	**event**				
	**(TB) censored**				
**Age**					
15–24	28	8,425	1	1	1
25–34	98	21,052	1.16 (0.75–1.74)	1.19 (0.78–1.81)	1.01 (0.28–3.70)
35–44	143	26,138	0.95 (0.63–1.42)	0.97 (0.65–1.46)	0.46 (0.12–1.75)
55–55	92	15,251	0.87 (0.57–1.33)	0.89 (0.58–1.36)	0.52 (0.13–2.11)
Above 55	44	7,477	0.75 (0.47–1.21)	0.78 (0.48–1.25)	0.60 (0.13–2.77)
**Sex**					
Male	188	21,795	1	1	1
Female	217	56,547	0.43 (0.36–0.52)	0.42 (0.34–0.50)	0.89 (0.41–1.92)
**CD4 categories**					
Below 350	42	7,766	1	1	1
350–500	6	2,555	0.41 (0.20–0.84)	0.42 (0.21–0.87)	0.19 (0.04–0.80)
Above 500	4	3,094	0.16 (0.06–0.40)	0.16 (0.07–0.42)	0.15 (0.04–0.64)
**Ever use art**					
No	51	5,356	1	1	1
Yes	354	72,987	0.46 (0.36–59)	0.43 (0.33–0.55)	0.34 (0.15–0.79)
**Functional status**					
Bedridden	58	2,589	1	1	1
Ambulatory	14	326	0.97 (0.56–1.71)	0.92 (0.52–1.63)	2.14 (0.23–19.94)
Working	327	74,594	0.05 (0.04–0.07)	0.05 (0.04–0.07)	0.15 (0.05-0.47)
**CTC enroll year**					
2003–2007	45	12,828	1	1	1
2008–2012	155	31,926	1.37 (1.03–1.84)	1.51 (1.12–2.03)	2.97 (1.05–8.43)
2013–2017	205	33,589	3.50 (2.66–4.59)	4.05 (3.04–5.39)	2.55 (0.79–8.20)
**Nutritional status**					
Ok	278	50,449	1	1	1
Moderate	45	3,558	5.53 (4.04–7.58)	6.94 (4.92–9.80)	0.93 (0.20–4.22)
Severe	11	207	27.95 (15.30–51.06)	28.05 (15.09–52.16)	9.27 (2.15–39.95)

After adjusting for both health facility type clusters and other important factors, only two factors were found to be significantly positively-related to incidence of TB, which are severe malnutrition, IRR 9.27 (95% CI 2.15–39.95), and being enrolled at a CTC between the years 2008 and 2012, IRR 2.97 (95% CI 1.05–8.43), compared to being enrolled in the years 2003 to 2007. The following factors remained as significantly protective against TB incidence among HIV patients after doing multilevel analysis. Having CD4 counts above 350 cells/μl with IRRs of 0.19 (95% CI 0.04–0.80) and 0.15 (95% CI 0.04–0.64) for CD4 counts between 350 and 500 cells/μl and above 500 cells/μl, respectively. Use of ART was protective by 66%, IRR 0.34 (95% CI 0.15–0.79), while a working functional status was protective against TB incidence by 85%, IRR 0.15 (95% CI 0.05–0.47) (Table 4). In this multilevel model and controlling for health facility types as variable clusters, the intercept variability across facilities was 0.78 (95% CI 0.23–2.73), with standard error (SE) of 0.5.

## Discussion

In this population of PLHIV attending CTCs for the years 2012 to 2017, in Northern Tanzania, the incidence rate of TB was 2.08 per 1,000 person-years, which is higher than the general TB incidence rate of 1.29 per 1,000 Tanzanian general population, for the year 2016, as reported by the Tanzania National TB and Leprosy Program (NTLP), but lower than the WHO TB incidence estimation of 2.7 per 1,000 population, in the year 2017 (7). But this number is within the estimated range of 1.5–4 new cases per 1,000 in most of the 30 high TB burden countries among the general population. Our estimated incidence rate is lower than other studies done in Tanzania, which showed an incidence of TB among HIV patients to be in the range of 8–17 per 1,000 person-years (15), but this was between the years of 2008 to 2010 for patients who were not on ART and before the introduction of IPT. Another study done in a major city in Tanzania, Dar es Salaam, found the incidence rate to be 27 per 1,000 person-years (13). The city is highly crowded, has a high TB diagnostic capacity and according to NTLP has the highest TB case notification rate in the country. In Nigeria, TB incidence was 5.7 per 1,000 person-years, among HIV patients on ARVs for the period of 2004 to 2012 (16). Higher incidences were found in South Africa (17) and Ethiopia (18) with TB incidences of 44 per 1,000 person-years and 86 per 1,000 person-years, respectively. According to WHO incidence rates need to be falling by 4–5% per year up to 2020, in order to reach the End TB Strategy milestone.

**Figure 1:**
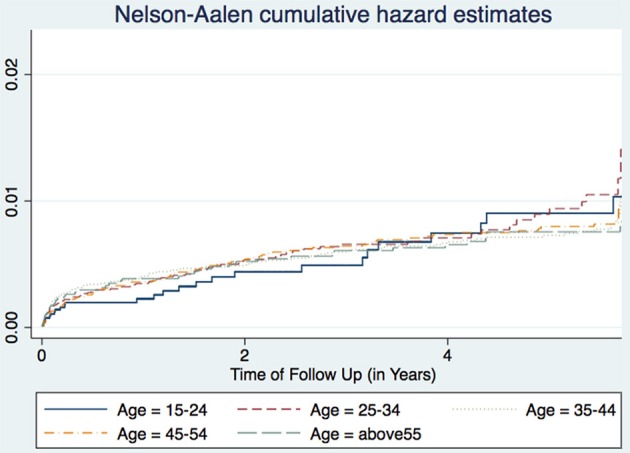
Cumulative hazard estimates (TB incidences) for age categories.

**Figure 2:**
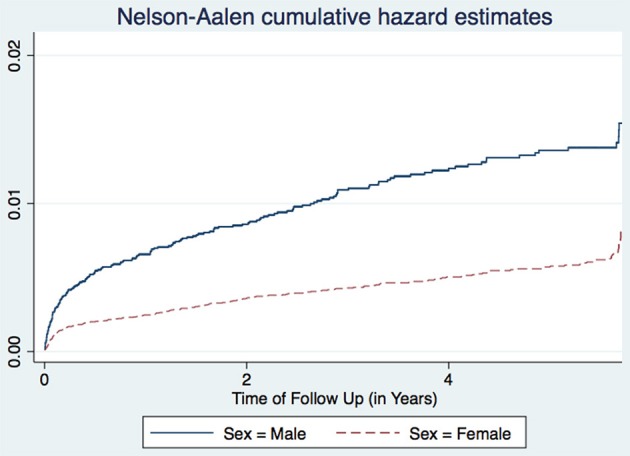
Cumulative hazard estimates (TB incidences) for sex.

A study in Nigeria (16) and another in South Africa (17), found males to have higher TB incidence than females. Age is also associated with TB incidence with patients aged 25–34 years having the highest incidence, while those aged 15–24 years having the lowest incidence trend (Figure 1). This agrees with other studies performed in Ethiopia (18) and Nigeria (16). Age and sex differences in TB incidence could be due to cultural factors and economical reasons, whereby men and those in the most economically productive age group are less likely to have time to attend clinics and to receive appropriate care, including Isoniazid Preventive Therapy, hence are more likely to be diagnosed with TB (Figure 2). Other studies have shown that males have an increased TB prevalence than females (especially in low- and middle-income countries) due to the fact that men are disadvantaged in seeking and/or accessing TB care in many settings (19). The same pattern has also been observed in Europe (20).

**Figure 3:**
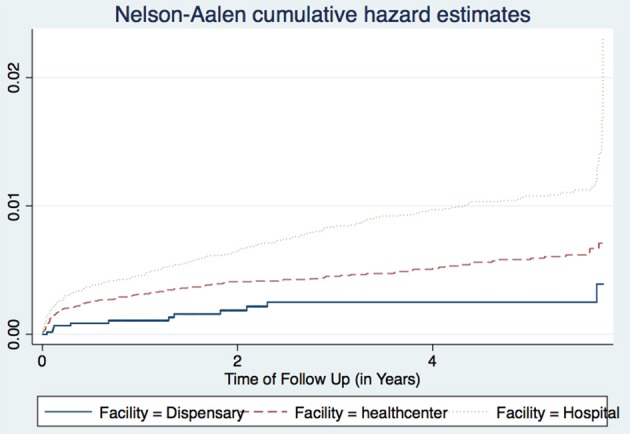
Cumulative hazard estimates (TB incidences) for health facility types.

The three regions of Arusha, Kilimanjaro, and Tanga had essentially similar TB incidence rates, and there were no significant differences between public and private facilities. All health facilities in this dataset had at least one TB case, and PLHIV seen at the hospitals for care and treatment had higher TB occurrence than those attending lower level facilities. This may be due to the fact that many lower level facilities have poor or inadequate availability of diagnostic equipment, and have low skilled health care workers. Hence leading to a reduced capacity for diagnosing TB as these co-infected patients tend to present with atypical TB manifestations which are difficult to diagnose. However, this could also have been due to the referral mechanisms, whereby most PLHIV with more advanced HIV and probably with active TB tend to be referred to hospitals for advanced care and treatment. In addition, most of these lower level facilities do not hospitalize their patients. This observation was more pronounced in the later years of the study, after the increased roll out of Genexpert MTB/RIF machines to many higher level facilities (Figure 3).

Severe malnutrition was found to be associated with an increased risk of TB among HIV patients in our study. Similar findings have been observed in other settings, especially among children (21) where malnutrition accounts for 26% of incident TB (22). It is also positively associated with TB progression (23), poor treatment outcomes (21) and delayed recovery (24). This is because it deteriorates the cell mediated and humoral immunity (21). Though most of the studies have focused on children, malnutrition is common among adult TB patients (25), as well as MDRTB patients (26) and in patients with other infections (27). Strategies to manage malnutrition should incorporate routine TB and HIV screening (28), and modifications to TB treatment (29), though some studies have found that these additional strategies are not yet effective (22) and that there is no significant association between nutritional status and TB severity (30). Even though this is controversial, but if implemented effectively, these strategies might prevent TB incidence and increase the probability of being cured (21, 31), and decrease the risk of TB mortality especially among children (32).

Incident TB increased almost three times for those enrolled in CTCs between 2008 and 2017 compared to those enrolled between 2003 and 2007, the reason for which could be that most of those enrolled between 2003 and 2007 were already on ARVs for some time when we started following up in 2012. In general TB incidence among HIV patients has been declining since 2012, with the exception being during 2017. This decline is congruent with the recent global and African data on TB epidemiological burden, that there has been a consistent decline in TB over the last decade, as well as in the most affected areas of Sub Saharan Africa (7). Success can be attributed to an improved health system; increased use of Isoniazid preventive therapy; increased HIV prevention and awareness (33); early TB diagnosis (11) and treatment; all of which reduces the risk of TB transmission, whilst also strengthening the collaboration between HIV and TB control activities when combined with other diseases. For the year 2017, which had a higher incidence of 3.54 per 1,000 person-years (Table 1), this could be attributed to the increased roll out of Genexpert MTB/RIF and that probability that most hospitals had these molecular tests installed.

Our findings have shown that a CD4 count above 350 cells/μl, or being on ARVs, was protective against the development of incident TB. HIV increases TB risk through increasing the risk of acquiring TB (3), or through reactivation of latent TB (2). Immunity against other infections including TB is compromised by HIV infection (34). The risk doubles after sero-conversion (5) and remains high within the first 3 years after enrollment at CTCs (18). TB risk is five times higher in the African region (1), and especially for those with a history of previous TB disease (15), and with a decreasing CD4 count (6), similar to our findings. As found in other studies (35), the risk of TB incidence is there even among HIV patients who are on IPT. The risk decreases with the use of ARVs (33) as our study has found but can still remain 5 times higher compared to HIV uninfected persons (36) despite an increase in their CD4 count. ART benefits are more pronounced in preventing TB in patients with a lower CD4 count (37), this is because ARVs in general restore immunity and so protect against developing TB, but this is controversial as other studies have found that ART use does not reduce TB incidence (38). Even so, this ART protection seems to be lost when one is on second line ARVs (39) as was also found in this study. Others have suggested controlling for hemoglobin levels as low hemoglobin can be used as a predictor of TB among these patients on ARVs (40).

Working functional status was significantly protective against TB incidence as found in other studies (53). Other studies found the risk factors for TB among HIV patients to be low hemoglobin (40), increased HIV viral load (41), genetics (42), WHO HIV stage 3, not taking Isoniazid (43), smoking, diabetes, alcohol use, crowded living, poverty (44), variation in compliance to taking ARTs (45), as well as social inequality in access to sanitation and health expenditure per capita. Other studies have shown that the following subpopulations have an increased risk of TB incidence: prisoners (46), migrants (47), health care workers (48), miners (4), and contacts of indexed TB cases (49). Other studies have found that other protective factors against TB incidence include the use of IPT (13, 50), cash transfers (51), as well as early TB detection and treatment initiation (52).

This study's strengths include the use of routinely collected data from a large number of patients and the inclusion of all health facilities providing electronic data for HIV care and treatment across the three regions. It has also assessed and adjusted for the differences between health facilities that might affect TB incidence. The study was limited in that only HIV positive patients who were identified to have a definitive diagnosis of TB, or to have started TB medications, during follow up were considered as TB incidence. The analysis has included all new cases of TB, including those with no microbiological TB confirmation. However, there could be an underestimation of new TB cases among HIV patients if there were no diagnostic tools available for presumptive TB cases (especially in lower level health facilities), or if there was a delay or gap between the diagnosis of TB and starting anti-TB medications. We recommend future studies on determining the mechanisms of nutritional effects on TB incidence and progression; the association between second line ARVs and TB incidence; and determining why the risk of TB remains higher even after improving the CD4 count of HIV patients on ART. We also recommend further analysis of the TB diagnosis cascade to see how effective the system is for TB diagnosis.

## Conclusion

Despite the prolonged decline of TB burden in most of the African countries, in the last decade TB is still a public health problem especially among HIV patients. Poor nutritional status and being enrolled at the CTCs after 2007 were significantly associated with TB incidence among HIV patients attending CTCs since 2003 in Tanzania. So there needs to be effective collaborative TB control strategies encompassing other diseases including HIV, and a continuing improvement of health system including the CTCs. Having a working functional status, a high CD4 count (above 350 cells/μl), and using ART were protective against TB incidence among HIV positive patients, though the use of second line ARVs was found to be a risk factor for developing TB. So all patients should be put on ARVs as soon as possible, and close monitoring of all patients with a CD4 count <350 cells/μl.

## Data Availability Statement

Study's data can be accessed from EM after permission and approval from the NACP and the Government of Tanzania.

## Author Contributions

EM, JT, MM, and SM designed the study and wrote the manuscript. EM, JT, and WM retrieved the data. EM and JT analyzed the data. All authors approved the final version of the manuscript.

### Conflict of Interest

The authors declare that the research was conducted in the absence of any commercial or financial relationships that could be construed as a potential conflict of interest.
